# Potassium Sodium Niobate-Based Lead-Free Piezoelectric Multilayer Ceramics Co-Fired with Nickel Electrodes

**DOI:** 10.3390/ma8115389

**Published:** 2015-11-03

**Authors:** Shinichiro Kawada, Hiroyuki Hayashi, Hideki Ishii, Masahiko Kimura, Akira Ando, Suetake Omiya, Noriyuki Kubodera

**Affiliations:** Murata Manufacturing Co., Ltd., Nagaokakyo, Kyoto 617-8555, Japan; hiroyuki_hayashi@murata.com (H.H.); hideki_ishii@murata.com (H.I.); mkimura@murata.com (M.K.); a_ando@murata.com (A.A.); sue_take@murata.com (S.O.); kubodera@murata.com (N.K.)

**Keywords:** ferroelectric material, potassium sodium niobate, (K,Na)NbO_3_, piezoelectric actuator, base metal inner electrode, multilayer structure

## Abstract

Although lead-free piezoelectric ceramics have been extensively studied, many problems must still be overcome before they are suitable for practical use. One of the main problems is fabricating a multilayer structure, and one solution attracting growing interest is the use of lead-free multilayer piezoelectric ceramics. The paper reviews work that has been done by the authors on lead-free alkali niobate-based multilayer piezoelectric ceramics co-fired with nickel inner electrodes. Nickel inner electrodes have many advantages, such as high electromigration resistance, high interfacial strength with ceramics, and greater cost effectiveness than silver palladium inner electrodes. However, widely used lead zirconate titanate-based ceramics cannot be co-fired with nickel inner electrodes, and silver palladium inner electrodes are usually used for lead zirconate titanate-based piezoelectric ceramics. A possible alternative is lead-free ceramics co-fired with nickel inner electrodes. We have thus been developing lead-free alkali niobate-based multilayer ceramics co-fired with nickel inner electrodes. The normalized electric-field-induced thickness strain (*S*_max_/*E*_max_) of a representative alkali niobate-based multilayer ceramic structure with nickel inner electrodes was 360 pm/V, where *S*_max_ denotes the maximum strain and *E*_max_ denotes the maximum electric field. This value is about half that for the lead zirconate titanate-based ceramics that are widely used. However, a comparable value can be obtained by stacking more ceramic layers with smaller thicknesses. In the paper, the compositional design and process used to co-fire lead-free ceramics with nickel inner electrodes are introduced, and their piezoelectric properties and reliabilities are shown. Recent advances are introduced, and future development is discussed.

## 1. Introduction

Piezoelectric ceramics are widely used in various devices, such as actuators, speakers, and transducers. Lead zirconate titanate (PZT)-based ceramics are usually used in piezoelectric devices [1,2]. A multilayer structure is used for those piezoelectric applications requiring large displacement or low-voltage driving, and a silver-palladium alloy is ordinarily used as the inner electrode material for PZT-based multilayer ceramics.

For barium titanate-based multilayer ceramic capacitors, nickel inner electrodes fired in a reducing atmosphere are used [3,4]. Nickel electrodes have many advantages, such as high electromigration resistance, high interfacial strength with ceramics, and greater cost effectiveness than silver palladium. However, PZT-based piezoelectric ceramics cannot be co-fired with nickel because the lead in PZT reduces easier than nickel, and PZT-based ceramics cannot be sintered in a reducing atmosphere, which does not oxidize nickel. On the other hand, it might be possible to co-fire lead-free piezoelectric ceramics with nickel in a reducing atmosphere.

Potassium sodium niobate (KNN)-based ceramics [5,6,7,8,9,10,11,12,13,14,15,16,17,18,19,20,21,22,23,24,25,26,27,28,29,30,31,32,33], bismuth sodium titanate-based ceramics [34,35,36,37], bismuth potassium titanate-based ceramics [37,38], ferroelectric ceramics with a tungsten bronze structure [39], and bismuth layer-structured ferroelectrics [40,41,42,43,44,45,46,47,48,49,50,51] have been extensively studied as lead-free piezoelectric ceramics. Ceramics containing bismuth cannot be co-fired with nickel as well as PZT-based ceramics because the bismuth reduces easier than nickel. Furthermore, KNN-based ceramics have a relatively high piezoelectric constant and Curie temperature in lead-free materials [5,6,7,8,9,10,11,12,13,14,15,16,17,18,19,20,21,22,23,24,25,26,27,28,29,30,31,32,33]. Therefore, we decided to use a potassium sodium niobate-based composition to develop multilayer piezoelectric ceramics with nickel inner electrodes.

In this review, our work on KNN-based multilayer piezoelectric ceramics co-fired with nickel inner electrodes is introduced. First, a KNN compositional study of firing in a reducing atmosphere is reviewed [52]. Then, a study on the reliability of KNN-based multilayer ceramics co-fired with nickel inner electrodes is described [53]. Next, a study on the feasibility of a KNN-based multilayer piezoelectric actuator co-fired with nickel inner electrodes is discussed. Finally, further progress in improving the piezoelectric properties of KNN-based ceramics fired in a reducing atmosphere is mentioned [54].

## 2. Fabrication of KNN-Based Multilayer Piezoelectric Ceramics Co-Fired with Nickel Inner Electrodes

In the first study reviewed, single-layer KNN-based samples without inner electrodes were fabricated to evaluate the reduction resistance [52]. (K,Na)NbO_3_–CaTiO_3_– and (K,Na)NbO_3_–CaZrO_3_–based compositions were selected because of their superior piezoelectric properties [27,28,29] Three powder samples composed of high-purity (99.9%) K_2_CO_3_, Na_2_CO_3_, Nb_2_O_5_, CaCO_3_, TiO_2_, ZrO_2_, and MnCO_3_ were prepared.

Sample KNN-CT-1: 0.96(K_0.5_Na_0.5_)NbO_3_ − 0.04CaTiO_3_
Sample KLN-CZ-1: 0.96(K_0.5_Na_0.5_)NbO_3_ − 0.04CaZrO_3_
Sample KNN-CZ-2: 0.96(K_0.5_Na_0.5_)NbO_3_ − 0.04CaZrO_3_ + 0.03Zr

**Figure 1:**
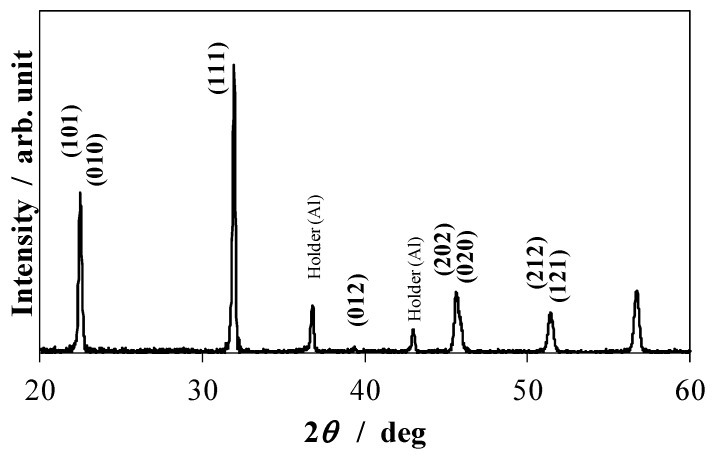
X-ray diffraction profile of KNN-CZ-2 powder calcined at 900 °C.

We added 5 mol % manganese to each sample as a sintering aid. The weighed powders were ball-milled with ethanol and calcined at 900 °C for 2 h in air. It was reported that calcination temperatures on the range from 700 °C to 850 °C produced well crystallized nanoparticles with a predominance of tetragonal phase and no presence of secondary phases for (K_0.44_Na_0.52_Li_0.04_)(Nb_0.86_Ta_0.10_Sb_0.04_)O_3_ [55]. However, in our study, the calcination temperature of 900 °C produced single phase particles and calcination temperatures below 900 °C produced secondary phases. It is thought that the difference of compositions causes the difference of calcination temperatures to obtain single phase particles. Figure 1 shows the X-ray diffraction profile of KNN-CZ-2 powder calcined at 900 °C.

The calcined powders were mixed with a 10 wt % polyvinyl acetate binder and ion exchange water to prepare a tape-casting slurry. The slurry was formed into green sheets using the doctor-blade technique, and the green sheets were stacked and pressed into green bodies, which were then formed into disks with a diameter of 10 mm and a thickness of 1 mm. The disks were fired at a rate of 0.5 °C/min from room temperature to 400 °C and held for 5 h in air to remove the binder. Then, the disks were fired a rate of 3 °C/min to 1080 °C and held for 2 h in a reducing atmosphere (oxygen partial pressure of 1 × 10^−11^ to 1 × 10^−10^ MPa). Silver electrodes were then sputtered on both surfaces of each sample. Finally, the samples were poled at an electric field of 3.0 kV/mm in silicon oil at 80 °C for 30 min.

The bulk densities were measured using the Archimedes’ method. The insulation resistance was measured at 100 V/mm with a digital electrometer (Advantest R8240). The dielectric constant, ε_33_^T^/ε_0_, was measured at a frequency of 1 kHz, and the radial-mode electromechanical-coupling factor, *k_p_*, was estimated using the resonance-antiresonance method with an impedance analyzer (Agilent 4294A). The piezoelectric constant, *d*_33_, was measured with a piezoelectric *d*_33_ meter (Piezotest PM300). The Curie temperature was determined from the temperature dependence of the dielectric constant.

The insulation resistivity and sintered body density of single-layer disk samples are listed in Table 1.

**Table 1:** Insulation resistivity and sintered body density of single-layer disk samples (measured temperature: 25 °C).

Composition	Insulation Resistivity (Ω·m)	Sintered Body Density (kg/m^3^) *
KNN-CT-1	7.7 × 10^4^	4.5 × 10^3^ (98%)
KNN-CZ-1	1.6 × 10^6^	4.0 × 10^3^ (87%)
KNN-CZ-2	6.3 × 10^8^	4.5 × 10^3^ (98%)

* Relative density is indicated in parentheses ( ).

After the KNN-CT-1 sample was fired in a reducing atmosphere, a high-density sintered body with a relative density of 98% was obtained, but the insulation resistivity was too low (7.7 × 10^4^ Ω·m) to enable polarization. Even though the sintered body density was sufficiently high, insulation resistivity was low. It is well known for BaTiO_3_, which is fired in a reducing atmosphere, that the Ti valence changes during the firing in a reducing atmosphere and the change of Ti valence creates the oxygen vacancies. Those oxygen vacancies cause a lower insulation resistance [56,57,58]. Therefore as same as BaTiO_3_, which is fired in a reducing atmosphere, the Ti valence may have changed during the firing in a reducing atmosphere and the insulation resistance became too low.

For this reason, the KNN-CZ-1 sample was fired in a reducing atmosphere in which the Ti, which has a fluctuating valence number, was replaced with Zr, which has a stable valence number. This resulted in higher insulation resistivity (1.6 × 10^6^ Ω·m), but it still was not high enough to enable polarization. This was because the sintered body relative density was only 87%, and the low insulation resistivity was apparently due to incomplete sintering. Various additives were examined for improving the sintering density of KNN-CZ-1, resulting in the KNN-CZ-2 composition in which 0.03 mol of ZrO_2_ was added to 1 mol of KNN-CZ-1. This improved the sintered body relative density to 98% and the insulation resistivity to 6.3 × 10^8^ Ω·m, sufficient to enable polarization. The piezoelectric properties of a KNN-CZ-2 single-layer disk sample are shown in Table 2. The piezoelectric constant, *d*_33_ was 160 pC/N.

**Table 2:** Piezoelectric properties of KNN-CZ-2 single-layer disk sample (measured temperature: 25 °C).

Composition	KNN-CZ-2
Grain size (μm)	0.7
Dielectric permittivity, ε_33_^T^/ε_0_	1180
Coupling coefficient, *k*_p_ (%)	31.8
Piezoelectric constant, *d*_33_ (pC/N)	160

**Figure 2:**
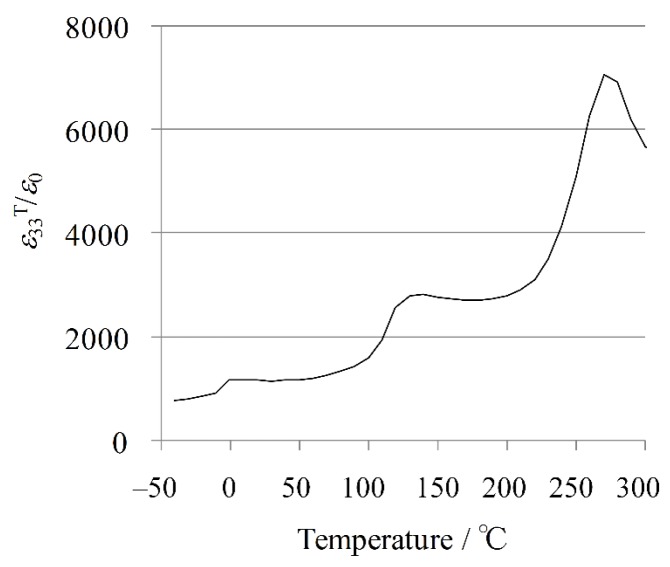
Temperature dependence of dielectric permittivity for KNN-CZ-2 single-layer sample.

The temperature dependence of the dielectric permittivity for the KNN-CZ-2 sample is plotted in Figure 2. It is well known that in KNN-based system there are four polymorphic phases, rhombohedral (R), orthorhombic (O), tetragonal (T), and cubic (C) phases with increasing temperature [59]. Thus, the transition temperatures are correspondingly defined as *T*_R-O_ (~0 °C), *T*_O-T_ (~140 °C), and *T*_C_ (~260 °C) from Figure 2.

Next, a multilayer KNN-CZ-2 sample was fabricated. A multilayer piezoelectric structure with 12 ceramic layers and 11 nickel inner electrodes was fabricated using the KNN-CZ-2 composition. Nickel paste was printed on green sheets fabricated as described above, which were then stacked and pressed into a green body, which was then fired in the same way as described above for the single-layer samples. The multilayer sample was sintered without warpage, and it turned dark brown due to the nickel inner electrodes, whereas the single-layer samples turned light brown. The obtained sample was cut by dicing, and external electrodes were formed by sputtering silver. The size of the sample was 18 mm (length) by 2 mm (width) by 0.4 mm (thickness). Its cross-section was observed with an optical microscope and its surface was observed with a scanning electron microscope (SEM). The electric-field-induced thickness strain was measured with a displacement meter (Mahr Millitron 1202D). The normalized strain (*S*_max_/*E*_max_) was calculated as the ratio of the maximum electric-field-induced thickness strain to the maximum electric field.

**Figure 3:**
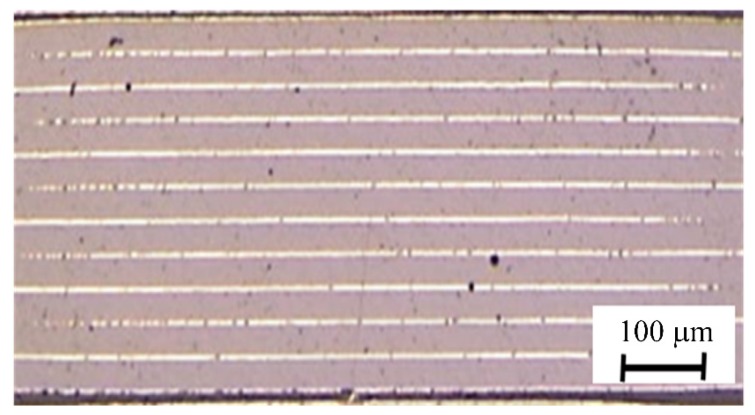
Optical microscope image of cross section of multilayer KNN-CZ-2 ceramic structure.

**Figure 4:**
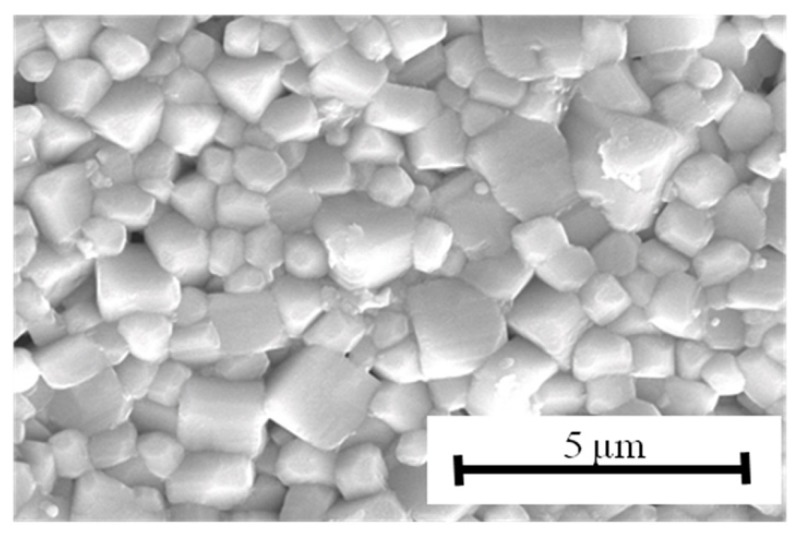
SEM micrograph of surface of multilayer KNN-CZ-2 sample.

An optical microscope image of a cross-section of the multilayer structure is shown in Figure 3. The thickness of each ceramic layer was about 35 μm, and the coverage of the nickel inner electrodes was high enough for practical use. The grain structure is shown in Figure 4. The grain size was about 0.7 μm; in other words, a fine-grain ceramic was obtained.

**Figure 5:**
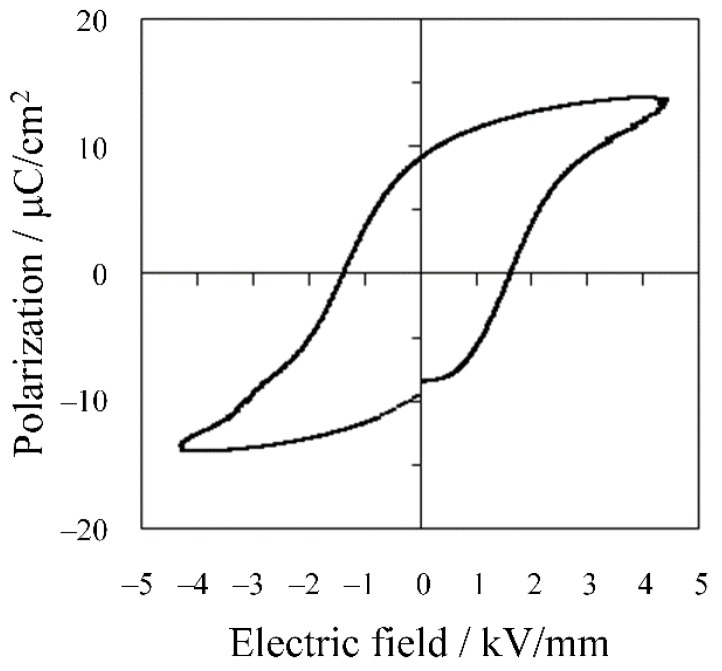
P-E hysteresis curve for multilayer KNN-CZ-2 sample.

The induced electric field dependence on polarization for the multilayer sample is shown in Figure 5. Typical ferroelectric polarization-electric field (P-E) hysteresis behavior was clearly observed, evidencing that the multilayer structure functioned normally. The remnant polarization and coercive field were respectively estimated to be 9.6 μC/cm^2^ and 1.5 kV/mm from this P-E hysteresis curve.

The strains and *S*_max_/*E*_max_ of the multilayer sample at electric fields of 1.0, 2.0, and 3.0 kV/mm are shown in Table 3. When the electric field was 2.0 kV/mm, *S*_max_/*E*_max_ was 360 pm/V, and when it was 1 or 3 kV/mm, *S*_max_/*E*_max_ was a bit lower and about the same value. The 360 pm/V is about twice the value of *d*_33_ for the single-layer sample, indicating that the contribution of domain-wall motion was probably larger than that of the PZT-based ceramics [60].

**Table 3:** Strains and *S*_max_/*E*_max_ of multilayer KNN-CZ-2 sample (measured temperature: 25 °C).

Induced Electric Field	1 kV/mm	2 kV/mm	3 kV/mm
Strain (%)	0.033	0.072	0.096
Normalized electric-field-induced thickness strain, *S*_max_/*E*_max_ (pm/V)	330	360	320

The *S*_max_/*E*_max_ value of 360 pm/V is about half that for the widely used PZT-based ceramic multilayer structures, which are usually stacked with silver palladium inner electrodes. However, the developed multilayer sample with nickel inner electrodes can achieve displacement comparable to that with PZT-based multilayer ceramics by stacking more ceramic layers that have smaller thickness. This is because the displacement is approximately proportional to the product of the ceramic layer thickness and applied electric field, which increases with a reduction in ceramic-layer thickness. Additionally, it becomes easier to reduce ceramic-layer thickness by using nickel electrodes rather than silver-palladium electrodes because nickel electrodes have high electromigration resistance and high interfacial strength with ceramics.

Thus, a KNN-based multilayer piezoelectric ceramic structure co-fired with nickel inner electrodes was successfully obtained by using a composition containing excess zirconium.

## 3. Reliability of KNN-Based Multilayer Piezoelectric Ceramics Co-Fired with Nickel Inner Electrodes

Environmental reliability is important for practical use. Since potassium and sodium oxides, the raw materials of KNN, are deliquescent materials, there are concerns about the reliability of KNN-based ceramics, especially under high-humidity conditions [6]. Several studies on the sinterability and reliability of KNN-based ceramics have been reported. For example, Takenaka and coworkers reported that dense and nondeliquescent KNbO_3_ (KN)-based ceramics were obtained using optimized calcination and milling processes [21,22,23,24]. Birol *et al.* reported that dense KN-based ceramics were realized by sintering in O_2_ atmosphere [61]. Furthermore, the humidity and thermal reliabilities of KNN-based ceramics have been reported [62,63].

We first investigated the relationship between excess zirconium and the behavior of the alkaline element of the composition by atomic absorption spectrometry. Then, the reliability of the resistance and piezoelectric properties of excess-zirconium-added KNN were studied under several environmental test conditions [53].

We prepared two sample structures of KNN-based multilayer ceramics with nickel inner electrodes. They had 11 layers, a length of 15 mm, a width of 2 mm, and a height of 0.5 mm.

Sample KNN-CZ-1ʹ: 0.98(K_0.5_Na_0.5_)NbO_3_ − 0.02CaZrO_3_
Sample KNN-CZ-3: 0.98(K_0.5_Na_0.5_)NbO_3_ − 0.02CaZrO_3_ + 0.03ZrO_2_

We added 5 mol % manganese to each sample as a sintering aid. A base composition of 0.98(K_0.5_Na_0.5_)NbO_3_ − 0.02CaZrO_3_ was selected to increase the Curie temperature.

The samples were fabricated using the process described above for the KNN-CZ-2 multilayer sample.

The insulation resistance and sintered body density of the two samples are listed in Table 4.

**Table 4:** Insulation resistivity and sintered body density of KNN-based multilayer samples (measured temperature: 25 °C).

Composition	Insulation Resistivity (Ω·m)	Sintered Body Density (kg/m^3^) *
KNN-CZ-1ʹ	1.6 × 10^6^	4.38 × 10^3^ (95%)
KNN-CZ-3	4.2 × 10^9^	4.50 × 10^3^ (98%)

* Relative density is indicated in parentheses ( ).

Both the density and resistivity of the KNN-CZ-3 sample were higher than those of the KNN-CZ-1ʹ one. The resistivity of KNN-CZ-1ʹ was insufficient for polarization. Therefore, the piezoelectric properties of KNN-CZ-1ʹ could not be measured in this study. The dielectric loss, tanδ, dielectric constant, ε_33_^T^/ε_0_, and electromechanical coupling coefficient, *k*_31_, were 3.6%, 660, and 22.8%, respectively.

The unreacted potassium of the calcined powder and the potassium vaporized during firing were estimated. In this experiment, the calcinations temperature was 900 °C. Water elutions of potassium and sodium were carried out to estimate the amount of unreacted alkaline oxides in the calcined powder. The calcined powder was put in a water base at 25 °C and stirred for 1 h. Then, the amounts of potassium and sodium eluted in the water were measured by atomic absorption spectrometry (AAS). The elution ratio is expressed as the weight ratio of the eluted amount to the weighed amount. The amount of vaporized alkaline elements was estimated by comparing the compositions of the presintered and after-sintered specimens by AAS. The vaporization ratio, *W*_vapor_, is expressed as the weight ratio and defined as

*W*_vapor_ = *W*_pre_ − *W*_after_ (1)

where *W*_pre_ and *W*_after_ are the weight ratios of the amount of alkaline elements for presintered and after-sintered specimens, respectively.

Table 5 shows the ratios of the estimated unreacted potassium and the vaporized potassium.

**Table 5:** Ratio of unreacted potassium in calcined powder and potassium vaporized during firing.

Composition	Unreacted Potassium (wt %)	Vaporized Potassium (wt %)
KNN-CZ-1ʹ	0.17	0.92
KNN-CZ-3	0.16	0.77

The unreacted and vaporized potassium ratios for the KNN-CZ-3 sample were lower than those for the KNN-CZ-1ʹ one. The amounts of unreacted and vaporized sodium were too small to be detected by AAS for both powders in this study. These results suggest that the excess zirconium accelerated the solid solution of potassium into the crystal lattice and prevented potassium evaporation. As the result, both the density and resistivity of the KNN-CZ-3 sample were apparently higher than those of the KNN-CZ-1ʹ one.

Reliability tests were carried out for KNN-CZ-3 sample using the test conditions shown in Table 6. The test samples were kept in a test chamber under these conditions for a given time range (from 0 to 500 h), and their resistivity and piezoelectric properties were measured after they were removed from the chamber. The samples were subjected to a heat cycle from −40 to 80 °C once every hour for the thermal shock test.

**Table 6:** Reliability test conditions.

Test name	Conditions
High-temperature test	85 °C
Low-temperature test	−40 °C
Humidity test	85 °C/85%RH
Thermal shock test	−40 to 85 °C (1 h/cycle)

**Figure 6:**
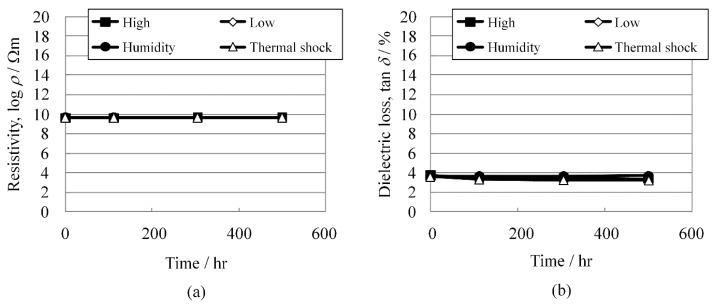
(**a**) Resistivity, log *ρ*; and (**b**) dielectric loss, tan *δ* change of KNN-CZ-3 under reliability test conditions.

As shown in Figure 6a,b, respectively, the resistivity changes and dielectric losses were extremely stable under these test conditions for at least 500 h.

**Figure 7:**
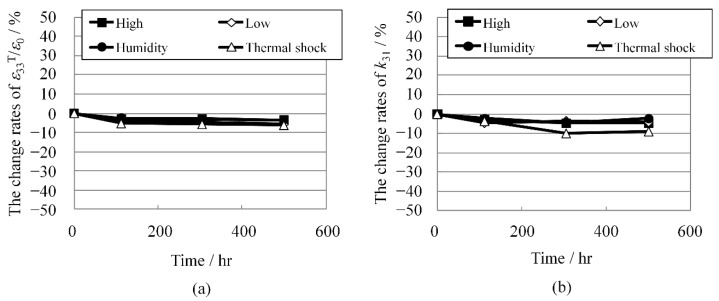
(**a**) Change rates of dielectric constant ε_33_^T^/ε_0_; and (**b**) change rates of electromechanical coupling coefficient *k*_31_ under reliability test conditions.

As shown in Figure 7a,b, respectively, the change rates of the dielectric constant and electromechanical coupling coefficient under the reliability test conditions were lower than 10% and comparable to those of ordinary PZT ceramics. The excess zirconium increased the resistivity and prevented potassium evaporation. The high resistivity should result in good piezoelectric properties, and the prevention of potassium evaporation should produce good reliability.

These results demonstrate that the reliability of a KNN-CZ-3 multilayer piezoelectric ceramic structure with nickel inner electrodes is good enough for practical application.

## 4. Feasibility of KNN-Based Multilayer Piezoelectric Actuator Co-Fired with Nickel Inner Electrodes

Before we fabricated a KNN-CZ-3 multilayer piezoelectric actuator with nickel electrodes, we measured the piezoelectric properties of a KNN-CZ-3 single-layer disk sample in the same way as used for the KNN-CZ-2 single-layer disk sample described above.

The piezoelectric properties of the sample are shown in Table 7. The piezoelectric constant of 130 pC/N is a little smaller than the 160 pC/N for the KNN-CZ-2 sample.

**Table 7:** Piezoelectric properties of KNN-CZ-3 single-layer disk sample *vs.* those of KNN-CZ-2 sample (measured temperature: 25 °C).

Composition	KNN-CZ-3	KNN-CZ-2
Grain size (μm)	0.7	0.7
Dielectric permittivity, ε_33_^T^/ε_0_	640	1180
Coupling coefficient, *k*_p_ (%)	34.0	31.8
Piezoelectric constant, *d*_33_ (pC/N)	130	160

**Figure 8:**
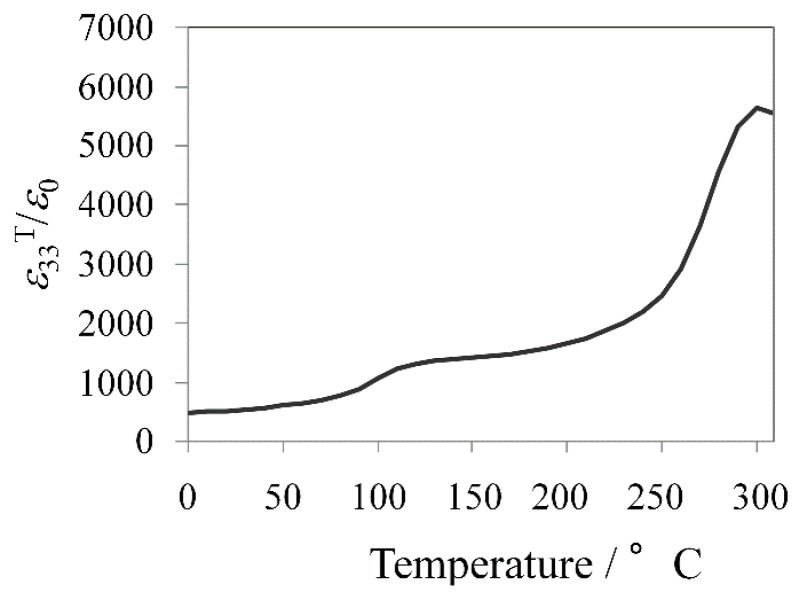
Temperature dependence of dielectric permittivity for KNN-CZ-3 single-layer sample.

The temperature dependence of the dielectric permittivity is plotted in Figure 8. There are four polymorphic phases, rhombohedral (R), orthorhombic (O), tetragonal (T), and cubic (C) phases with increasing temperature in KNN-based system, mentioned above [56]. Thus, the transition temperatures are correspondingly defined as *T*_O-T_ (at ~110 °C) and *T*_C_ (at ~300 °C) from Figure 8.

These results show that the KNN-CZ-3 sample exhibited suitable piezoelectric properties for a piezoelectric actuator. We thus fabricated a 101-layer piezoelectric actuator by using the KNN-CZ-3 composition and the same process used for the multilayer ceramic structure with nickel electrodes described above.

**Figure 9:**
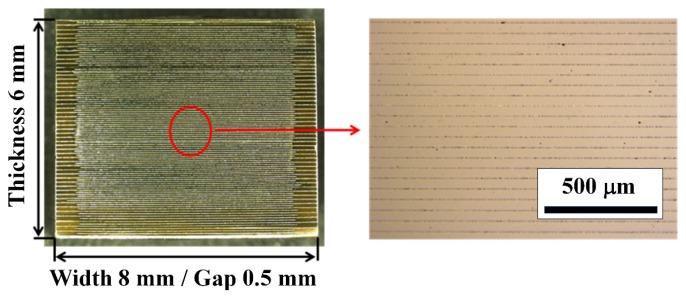
Optical microscope image of cross section of KNN-CZ-3 multilayer actuator.

Figure 9 shows an optical microscope image of a cross-section of the KNN-CZ-3 multilayer actuator. The thickness of ceramic layer was about 60 μm, and the coverage of the nickel inner electrodes was high enough for practical use.

**Figure 10:**
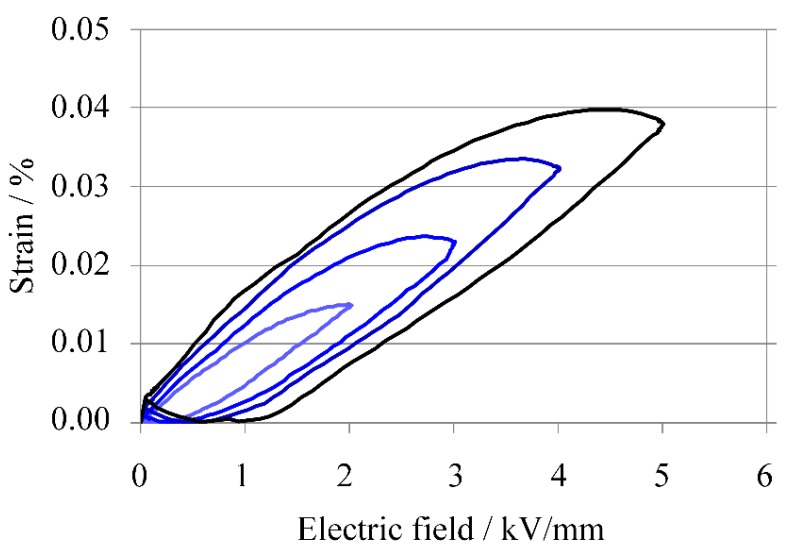
Strain-electric field curves of KNN-CZ-3 multilayer actuator.

At an electric-field of 5 kV/mm, *S*_max_/*E*_max_ was 80 pm/V, and when the electric field was from 2 to 5 kV/mm, *S*_max_/*E*_max_ was about the same. This value of 80 pm/V is much lower than the 360 pm/V of the KNN-CZ-2 multilayer sample, which had 11 ceramic layers. It is thought that disconnections occurred at the connection electrode when a high electric field was applied to the 101-layer sample because the strain after applying a high electric field was much smaller than that before applying the field, as evident in Figure 10.

The strain-electric-field curves are shown in Figure 10.

Although this developed KNN-CZ-3 multilayer ceramic structure is possibly suitable for practical use, more development work is needed before it is ready for practical application.

## 5. Further Progress in Improving Piezoelectric Properties of KNN-Based Ceramics Fired in a Reducing Atmosphere

It has been reported that the Curie temperature of (Ba,Ca)TiO_3_ ceramics can be increased by doping Sn^2+^ in the A-site of perovskite structures by calcining and sintering in a reducing atmosphere [64,65,66,67]. We investigated the Curie temperature and piezoelectric properties of Sn^2+^-doped KNN-based ceramics that were calcined and sintered in a reducing atmosphere [60].

We chose KNN-CZ-3 and (K,Na)NbO_3_ + SnO + ZrO_2_, which was obtained by doping Sn^2+^ instead of Ca^2+^. We then prepared KNN-based single-layer disk samples without inner electrodes.

Sample KNN-CZ-3: 0.98(K_0.5_Na_0.5_)NbO_3_ − 0.02CaZrO_3_ + 0.03ZrO_2_
Sample KNN-SZ: 0.98(K_0.5_Na_0.5_)NbO_3_ − 0.02SnZrO_3_ + 0.03ZrO_2_

We added 5 mol % manganese to each sample as a sintering aid. These weighed powders were composed of high-purity (99.9%) K_2_CO_3_, Na_2_CO_3_, Nb_2_O_5_, CaCO_3_, SnO, and MnCO_3_ and were ball-milled with ethanol and calcined at 900 °C for 2 h in a reducing atmosphere (oxygen partial pressure of 1 × 10^−16^ MPa). After calcination, KNN-CZ-3 and KNN-SZ single-plate disk samples were fabricated using the same process used for the KNN-CZ-2 single-plate disk sample described above.

The resistivities and densities of the two samples are shown in Table 8. The insulation resistivities were high enough for polarization.

**Table 8:** Insulation resistivity and sintered body density of multilayer Sn^2+^-doped KNN-based ceramic samples (measured temperature: 25 °C).

Sample	Insulation Resistivity (Ω·m)	Sintered Body Density (kg/m^3^) *
KNN-CZ-3	1.5 × 10^9^	4.21 × 10^3^ (92%)
KNN-SZ	1.9 × 10^9^	4.47 × 10^3^ (97%)

* Relative density is indicated in parentheses ( ).

**Figure 11:**
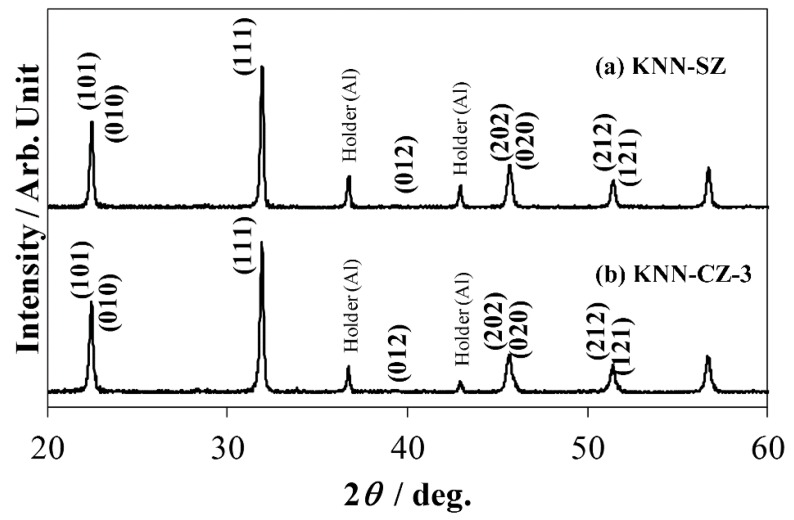
(**a**) X-ray diffraction profile of KNN-SZ ceramics; and (**b**) X-ray diffraction profile of KNN-CZ-3 ceramics.

X-ray diffraction (XRD) patterns of KNN-CZ-3 and KNN-SZ are shown in Figure 11. The crystal structures of KNN-CZ-3 and KNN-SZ were orthorhombic.

The piezoelectric properties of the KNN-CZ-3 sample, which contained powder calcined in air, and the KNN-CZ-3 and KNN-SZ samples, which contained powder calcined in reducing atmosphere, are shown in Table 9.

**Table 9:** Piezoelectric properties of single-layer disk samples (measured temperature: 25 °C).

Composition	KNN-CZ-3	KNN-CZ-3	KNN-SZ
Calcination condition	In air	In reducing atmosphere	
Dielectric permittivity, ε_33_^T^/ε_0_	640	684	1517
Coupling coefficient, *k*_p_ (%)	34.0	27.1	31.9
Piezoelectric constant, *d*_33_ (pC/N)	130	130	190
Curie temperature, *T*_C_ (°C)	300	300	300

The dielectric constant of the KNN-SZ sample, 1517, was much higher than those of the KNN-CZ-3 samples. Its piezoelectric constant, 190 pC/N, was about 1.5 times that of the KNN-CZ-3 samples. In the case of KNN-CZ-3, there are not so much differences between the calcination in air and the calcination in a reducing atmosphere. Therefore these results are not caused by only the calcinations in a reducing atmosphere, but also by doping Sn instead of Ca. This result suggests that the point of enhancing piezoelectric properties is doping Sn^2+^ in the A-site of perovskite structures by calcining and sintering in a reducing atmosphere, because Sn^2+^ easily changes to Sn^4+^ by calcining and sintering in air, and the ionic radius of Sn^4+^ is too small to dope Sn in A-site of perovskite structures.

The enhancement of piezoelectric properties is attributed to the phase transition behaviors. The Curie temperatures of the KNN-CZ-3 and KNN-SZ samples were all 300 °C while the tetragonal-orthorhombic phase transition temperature (*T*_O-T_) of the KNN-SZ sample was 90 °C, 60 °C lower than that of the KNN-CZ-3 samples. This shift in *T*_O-T_ due to doping Sn in the A-site of perovskite structures instead of Ca is probably what caused the increase in the piezoelectric properties, *k*_p_ and *d*_33_ [60].

These results indicate that the piezoelectric *d* constant of a KNN-based multilayer ceramic structure with nickel inner electrodes can be increased by doping with Sn^2+^ instead of Ca^2+^.

## 6. Conclusions

We have reviewed our work on the development of KNN-based piezoelectric multilayer ceramic structures co-fired with nickel electrodes. The *S*_max_*/E*_max_ of a KNN-based multilayer piezoelectric sample co-fired with nickel electrodes was about half that for the widely used PZT-based ceramic multilayer structure, which is usually stacked with silver palladium inner electrodes. However, the developed multilayer sample with nickel inner electrodes can achieve comparable displacement by stacking more ceramic layers with less thickness. This is because the displacement is approximately proportional to the product of the ceramic layer thickness and applied electric field, which increases with a reduction in the ceramic-layer thickness. Additionally, it is easier to reduce the ceramic-layer thickness by using nickel electrodes rather than silver-palladium ones because nickel electrodes have high electromigration resistance and high interfacial strength with ceramics. The reliability of the KNN-based multilayer piezoelectric sample co-fired with nickel electrodes was comparable to that of ordinary PZT ceramics. We fabricated a KNN-CZ-3 multilayer actuator, but its strain property was not good enough for practical use. It is thought that disconnections occurred at the connection electrode when a high electric field was applied to the 101-layer sample. Therefore, more development work is needed to make it suitable for practical use.

Different techniques for co-firing alkali niobate based-ceramics with a base metal have recently been proposed [68,69,70,71,72,73]. Furthermore, studies of multilayer KNN-based lead-free piezoelectric ceramics with silver-palladium electrodes have been reported [74,75]. Moreover, many studies on the application of KNN-based ceramics and improving the piezoelectric properties of KNN-based ceramics have been reported [76,77,78,79,80,81,82,83,84,85,86,87,88,89]. These activities indicate that a KNN-based multilayer ceramic structure is widely considered to be a good candidate for lead-free piezoelectric devices.

It is thus concluded that the developed KNN-based multilayer ceramic structure with nickel inner electrodes is a promising candidate for use in lead-free piezoelectric devices.
